# Development and Evaluation of a Shrimp Virus (IHHNV)-Mediated Gene Transfer and Expression System for Shrimps

**DOI:** 10.3390/ijms25168999

**Published:** 2024-08-19

**Authors:** Yiwen Tao, Jinwu Wang, Rui Xiao, Qingli Zhang, Huarong Guo

**Affiliations:** 1 Key Laboratory of Marine Genetics and Breeding (Ministry of Education), College of Marine Life Sciences, Ocean University of China, Qingdao 266003, China; eventy5@163.com (Y.T.); 15684726605@163.com (J.W.); xr_1310839936@163.com (R.X.); 2 Key Laboratory of Evolution & Marine Biodiversity (Ministry of Education), Institute of Evolution & Marine Biodiversity, Ocean University of China, Qingdao 266003, China; 3 Yellow Sea Fisheries Research Institute, Chinese Academy of Fishery Sciences, Qingdao 266071, China; zhangql@ysfri.ac.cn

**Keywords:** shrimp, infectious hypodermal and hematopoietic necrosis virus (IHHNV), virus-mediated gene transfer and expression system, viral packaging, shrimp hemolymph cells, insect Sf9 cells

## Abstract

An efficient gene transfer and expression tool is lacking for shrimps and shrimp cells. To solve this, this study has developed a shrimp DNA virus-mediated gene transfer and expression system, consisting of insect Sf9 cells for viral packaging, the shrimp viral vector of pUC19-IHHNV-PH-GUS and the baculoviral vector of Bacmid or Bacmid-VP28 encoding the shrimp WSSV envelope protein VP28. The pUC19-IHHNV-PH-GUS vector was constructed by assembling the genomic DNA of shrimp infectious hypodermal and hematopoietic necrosis virus (IHHNV), which has shortened inverted terminal repeats, into a pUC19 backbone, and then an expression cassette of baculoviral polyhedron (PH) promoter-driven *GUS* (β-glucuronidase) reporter gene was inserted immediately downstream of IHHNV for proof-of-concept. It was found that the viral vector of pUC19-IHHNV-PH-GUS could be successfully packaged into IHHNV-like infective virions in the Sf9 cells, and the gene transfer efficiency of this system was evaluated and verified in three systems of Sf9 cells, shrimp hemolymph cells and tissues of infected shrimps, but the GUS expression could only be detected in cases where the viral vector was co-transfected or co-infected with a baculovirus of Bacmid or Bacmid-VP28 due to the Bacmid-dependence of the PH promoter. Moreover, the packaging and infection efficiencies could be significantly improved when Bacmid-VP28 was used instead of Bacmid.

## 1. Introduction

Penaeid shrimp is an economically important aquaculture species in China. However, an efficient gene transfer and expression tool is lacking for both shrimp individuals and in vitro cultured shrimp cells. At the very beginning, many attempts had been made to introduce foreign genes into the eggs or early embryos of penaeid shrimp by physical methods like microinjection, electroporation and particle bombardment, but these attempts failed [1,2,3]. This can be attributed to the inherent problems, including the injection-induced burst, especially for homolecithal eggs, the quick cleavage, and the low integration and survival rates. Later, chemical methods like lipofection and biological methods of virus-mediated gene transfer and expression systems, including mammalian-sourced modified retrovirus, lentivirus and adeno-associated virus (AAV), as well as insect-sourced modified baculovirus, were successively tried in in vitro cultured shrimp cells, but they achieved limited success, with very low transfection or infection efficiencies in comparison with the results from mammalian tumor cells or insect cells due to the lack of actively dividing shrimp cells and the extremely low tropism of the viruses used [4,5,6,7,8,9]. Thus, an efficient gene transfer and expression tool is urgently needed for shrimps and shrimp cells.

It has been well recognized that a lot of great success in the biological field can be attributed to a great degree to the use of mammalian virus-mediated gene transfer and expression systems such as retrovirus, lentivirus, adenovirus and AAV because of their high infectivity and high gene delivery efficiency as well as their low cytotoxicity [10,11,12,13]. However, all of the aforementioned mammalian virus-mediated expression systems had extremely low tropism in shrimp cells, even though they were improved to be pantropic by pseudo-typing with a foreign envelope glycoprotein of vesicular stomatitis virus (VSV-G) [4,5,6,7,8,14]. For example, Pu et al. [4] and Chen et al. [5] further improved the tropism of the pantropic retrovirus and lentivirus expression systems in shrimp cells by introducing two envelope proteins of VP28 and VP19 of shrimp white spot syndrome virus (WSSV) into the envelope of the corresponding virions packaged via the co-transfection method, respectively; however, the infection and expression efficiencies obtained in shrimp cells were far lower than what was achieved in mammalian tumor cells. Recently, Tao et al. [8] modified the capsid of the packaged AAV-2 by introducing the shrimp WSSV-sourced tegument protein of VP26 or shrimp infectious hypodermal and hematopoietic necrosis virus (IHHNV)-sourced capsid protein (IHCP) via the co-transfection method and significantly improved the tropism of the modified AAV-2 in shrimp cells, but the infection and expression efficiencies obtained in shrimp cells were still much lower than those in mammalian cells. 

Attempts have also been made in terms of the improvement of Bac-to-Bac insect baculovirus expression systems since unmodified baculovirus could not efficiently infect shrimps and shrimp cells [9,15]. For example, Puthumana et al. [15] modified the baculovirus vector by insertion of shrimp virus-sourced promoters of Ie1 (WSSV) and P2 (IHHNV) and found that the modified baculovirus could successfully infect the adult shrimp tissues, although the infection efficiency was still low (<20%). In contrast, Wu et al. [9] developed a shrimp WSSV envelope protein VP28-pseudo-typed baculovirus expression system and found that the improved baculovirus could infect the primarily cultured shrimp hemolymph cells at a very low efficiency (1.2%) but adult shrimp tissues at an extremely high efficiency of nearly 100%, suggesting the better performance of the insect baculovirus in shrimps in contrast to mammalian viruses [4,5,6,7]. 

Compared with mammalian and insect viruses, shrimp virus has an incomparable advantage in the tropism to shrimp cells. Thus, we believe that efficient infection and foreign gene expression in shrimp cells can be expected from the development of a shrimp virus-mediated gene transfer and expression system. Recently, based on a shrimp RNA virus of *Macrobrachium rosenbergii* nodavirus (MrNV), Alenton et al. [16] developed a shrimp viral vector, a replication-incompetent mutant MrNV(ΔRdRp)-GFP, for RNA delivery, paving the way for the oral delivery of antiviral therapeutics in farmed crustaceans. However, to date, no shrimp DNA virus-mediated gene transfer and expression system have ever been established.

The infectious hypodermal and hematopoietic necrosis virus (IHHNV) belongs to the genus Penstylhamaparvovirus, family Parvoviridae and subfamily Hamaparvovirinae. It was also called PstDV because it was first found from *Penaeus stylirostris* in Hawaii (USA) [17,18], and later found in other shrimp species, crabs and bivalves [19,20,21,22,23]. IHHNV was found to be an unenveloped icosahedral DNA virus containing a linear single-stranded genomic DNA of no more than 4.1 kb in size, the smallest known penaeid shrimp virus [24]. This makes it feasible for the genomic DNA of shrimp IHHNV to be engineered into an expression vector. The genomic DNA of IHHNV contains three open reading frames of ORF1, ORF2 and ORF3, driven by three promoters of P2, P11 and P61 in their upstream, respectively [25,26]. These three promoters were found to be active not only in shrimp cells but also in insect and fish cells. Moreover, the capsid protein (ORF3) of IHHNV had been over-expressed in bacterial or insect cells and then self-assembled in vitro into virus-like particles (VLPs) and successfully used as a nanocarrier to deliver foreign genes into shrimp cells [27,28,29]. However, the use of VLPs as a gene delivery tool is limited due to the high cost and extensive labor involved in the production of VLPs and the relatively low DNA-loading efficiency. 

Suitable packaging cells are essential for a virus-mediated expression system. In consideration of the lack of an immortalized shrimp cell line and the accumulated evidence of the infectivity of several shrimp viruses in insect cells [30,31,32,33,34] and the successful propagation of IHHNV in shrimp–insect hybrid cells of PmLyO-Sf9 [35], in this study, the insect Sf9 cell line was chosen as a packaging cell line for the IHHNV-based expression vector, although electron microscopy evidence on the formation of shrimp viral particles in the infected insect cells has not yet been reported.

This study aims to develop a shrimp virus (IHHNV)-mediated gene transfer and expression system using insect Sf9 cells as packaging cells. To achieve this, the near full-length genomic DNA of shrimp IHHNV had been isolated and then inserted into a prokaryotic pUC19 backbone to obtain the cyclized plasmid of pUC19-IHHNV. After that, an IHHNV-based expression vector was constructed by inserting an expression cassette of PH-GUS-SV40 pA, the baculoviral polyhedron (PH) promoter-driven *GUS* (β-glucuronidase) gene, into pUC19-IHHNV immediately downstream of IHHNV and then packaged into IHHNV-like viral particles in the insect Sf9 cells by lipofection. And then the gene transfer and expression efficiency of this shrimp IHHNV-mediated expression system were evaluated and further improved in three systems of insect Sf9 cells, shrimp hemolymph cells and adult tissues of infected shrimps via the expression of the *GUS* reporter gene for proof-of-concept. 

## 2. Results 

### 2.1. Cloning and Cyclizing of the Genomic DNA of Shrimp IHHNV

**Figure 1:**
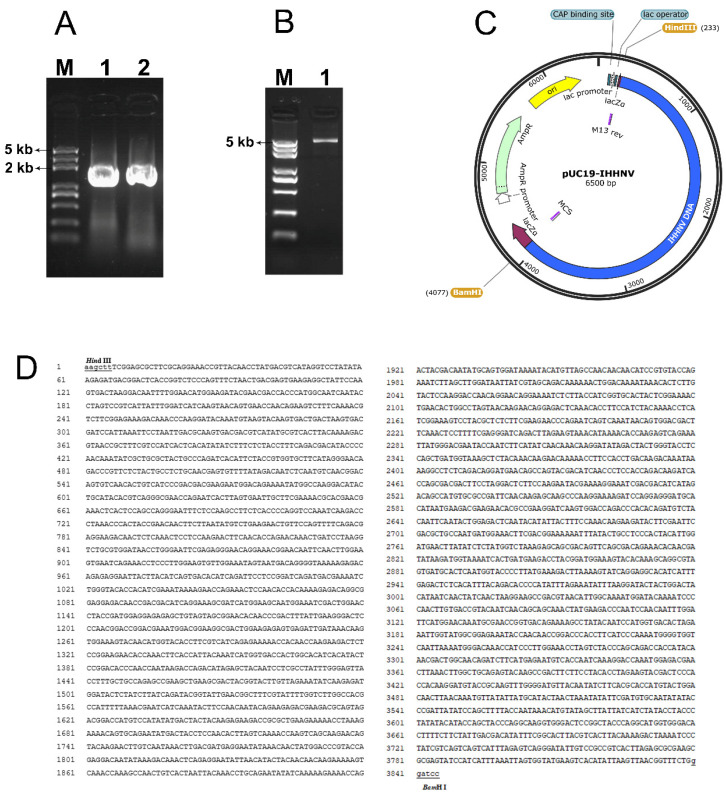
Plasmid construction of pUC19-IHHNV by cloning and inserting the genomic DNA of the shrimp IHHNV into a prokaryotic cloning plasmid of pUC19 via a head-to-tail linkage. (**A**) The agarose gel electrophoresis result of the two genomic fragments amplified from the anterior and posterior part of the genomic DNA of shrimp IHHNV, respectively. Lane 1, the anterior fragment of 1–1910 bp. Lane 2, the posterior fragment of 1870–3833 bp. Lane M, DNA Marker. (**B**) The agarose gel electrophoresis result of the cyclized plasmid of pUC19-IHHNV. Lane 3, pUC19-IHHNV. (**C**) The cyclized plasmid map of pUC19-IHHNV (6.5 kb). (**D**) The sequencing result of the cyclized plasmid of pUC19-IHHNV. The restriction endonuclease sites are underlined.

As shown in Figure 1, two overlapped fragments, located at positions 1–1910 bp and 1870–3833 bp in the isolated genomic DNA of shrimp IHHNV, had been successfully amplified from the total genomic DNAs of IHHNV-infected shrimps and sequenced (Figure 1A). Moreover, the two overlapped IHHNV fragments were head-to-tail jointed together and connected with the *Hind* III- and *Bam*H I- linearized pUC19 vector correctly, and thus a circular plasmid, named pUC19-IHHNV, was successfully constructed, consisting of a genomic DNA of shrimp IHHNV, which had shortened inverted terminal repeats (ITR) in its both ends, and a prokaryote cloning vector pUC19 (Figure 1B–D). The genomic DNA of shrimp IHHNV obtained was 3833 bp and that of pUC19 was 2.7 kb in size; thus, the plasmid of pUC19-IHHNV derived from the head-to-tail connection of pUC19 and IHHNV is 6.5 kb in size. It has also been found that the newly constructed plasmid of pUC19-IHHNV could be transformed into competent *E. coli* cells and multiplied within the bacterial cells; in other words, the successful construction of pUC19-IHHNV made it possible that the genomic DNA of shrimp IHHNV can be duplicated in bacterial cells (Figure 1B–D). 

### 2.2. Construction of Shrimp IHHNV-Based Expression Vector of pUC19-IHHNV-PH-GUS

**Figure 2:**
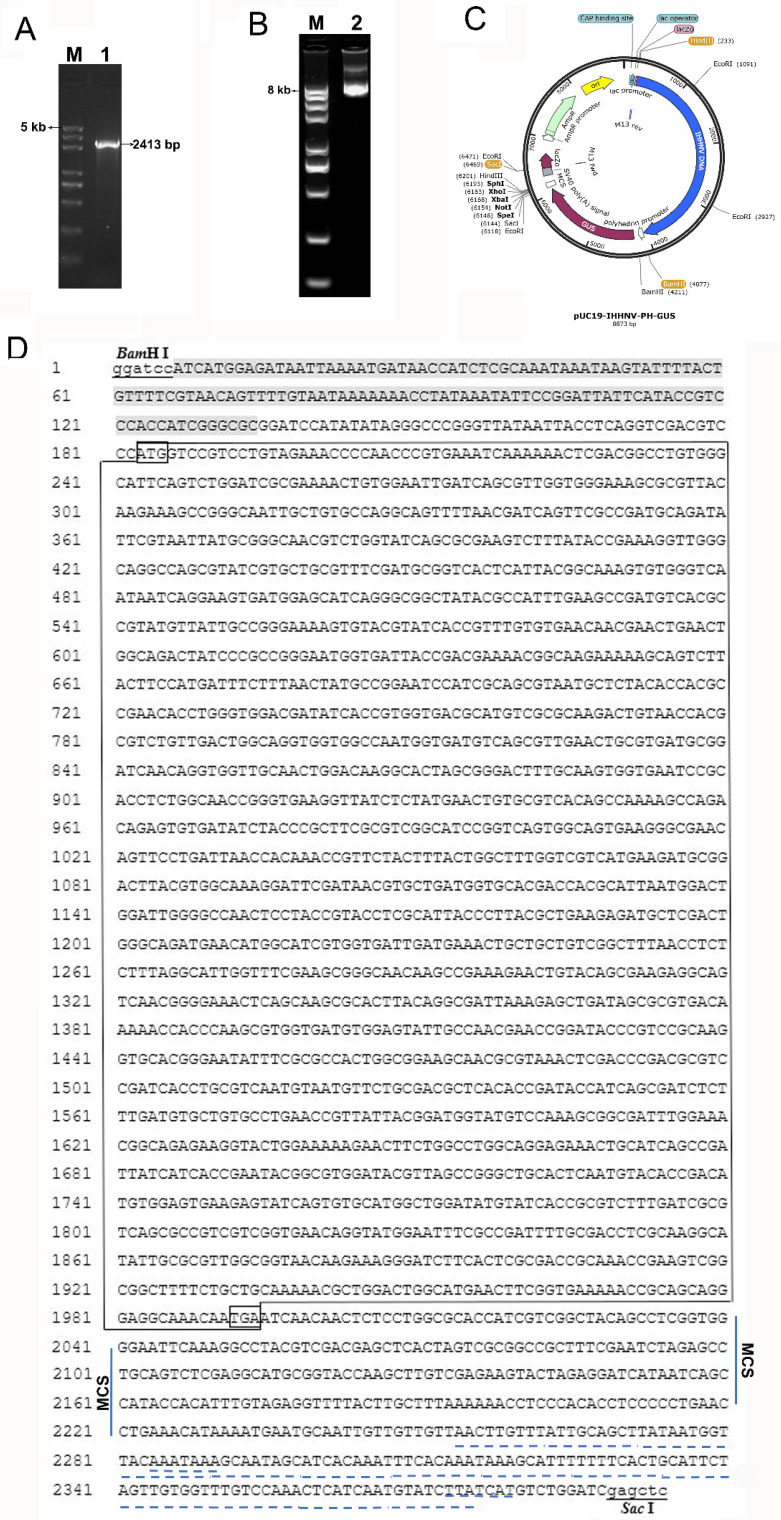
Construction of the viral expression vector of pUC19-IHHNV-PH-GUS by inserting an exogenous concatemer of PH-GUS-MCS-SV40 pA into the cyclized plasmid of pUC19-IHHNV. (**A**) The agarose gel electrophoresis result of the PCR product of PH-GUS-MCS-SV40 pA carrying a 30 bp pUC19-IHHNV-sourced homology arm in both ends. Lane 1, PH-GUS-MCS-SV40 pA. Lane M, DNA Marker. (**B**) The agarose gel electrophoresis result of the viral expression plasmid of pUC19-IHHNV-PH-GUS. Lane 2, pUC19-IHHNV-PH-GUS (8.9 kb). (**C**) The plasmid map of pUC19-IHHNV-PH-GUS. (**D**) The sequencing result of the expression plasmid of pUC19-IHHNV-PH-GUS. The restriction endonuclease sites of *BamH* I and *Sac* I are lowercase and underlined. The sequence of the PH promoter is shaded in a gray color. The sequence of the *GUS* gene is boxed in a larger size, and the start and stop codons are boxed in a smaller size. The terminator of SV40 pA is underlined with a dotted line and the poly(A) signal is double underlined with a dotted line. The multiple cloning site (MCS) is indicated in vertical lines.

As shown in Figure 2, a viral expression vector of pUC19-IHHNV-PH-GUS was successfully constructed by inserting an exogenous expression cassette of PH-GUS-MCS-SV40 pA (i.e., PH-GUS in abbreviation) into the *BamH* I and *Sac* I sites of the cyclized plasmid of pUC19-IHHNV. The PH-GUS-MCS-SV40 pA fragment was composed of an insect baculovirus-sourced promoter of PH (polyhedron), a reporter gene of GUS (β-glucuronidase), an MCS (multiple cloning site) and a transcriptional terminator of SV40 pA. It is 2413 bp in size, with a 30 bp pUC19-IHHNV-sourced homologous arm included in both ends. Thus, the shrimp IHHNV-based expression vector of pUC19-IHHNV-PH-GUS is 8.9 kb in size and can be used for the expression of the *GUS* gene and/or other genes in insect cells due to the presence of the PH promoter-driven *GUS* gene and the associated multiple cloning sites (MCS). The PH promoter was chosen here because of its high activity in Sf9 cells. 

### 2.3. GUS Expression of Shrimp IHHNV-Based Expression Vector of pUC19-IHHNV-PH-GUS in Sf9 Cells Was Dependent on the Presence of Insect Baculovirus of Bacmid or Bacmid-VP28

**Figure 3:**
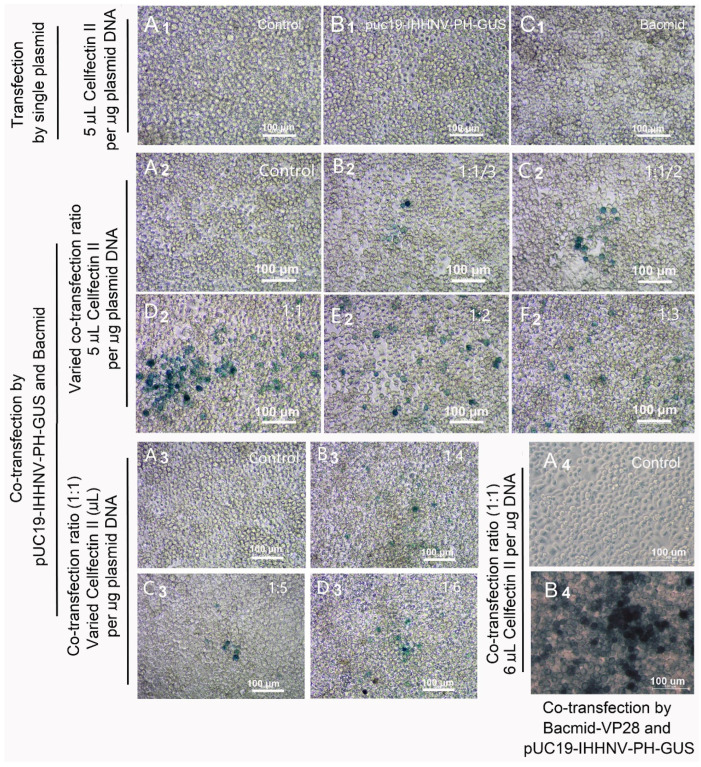
GUS expression of the shrimp IHHNV-based expression vector of pUC19-IHHNV-PH-GUS in Sf9 cells was dependent on the presence of insect baculovirus of Bacmid or Bacmid-VP28. The expression of the *GUS* gene was detected by X-gluc staining at 120 h post transfection. (**A1**–**A4**) The un-transfected Sf9 cells (control). (**B1**) The Sf9 cells transfected by pUC19-IHHNV-PH-GUS. (**C1**) The Sf9 cells transfected by Bacmid. (**B2**–**F2**) The Sf9 cells co-transfected by pUC19-IHHNV-PH-GUS and Bacmid at varying co-transfection ratios of 1:1/3 (**B2**), 1:1/2 (**C2**), 1:1 (**D2**), 1:2 (**E2**) and 1:3 (**F2**) but at the same transfection reagent ratio of 5 μL Cellfectin II per μg DNA. Panels (**B3**,**C3**,**D3**) are the Sf9 cells co-transfected by pUC19-IHHNV-PH-GUS and Bacmid at varying transfection reagent ratio of 4 (**B3**), 5 (**C3**) and 6 (**D3**) μL Cellfectin II per μg DNA but at the same co-transfection ratio of 1:1. (**B4**) The Sf9 cells co-transfected by pUC19-IHHNV-PH-GUS and Bacmid-VP28 at the optimized co-transfection ratio of 1:1 and transfection reagent ratio of 6 μL Cellfectin II per μg DNA. Scale bar, 100 μm.

As shown in Figure 3(B1), it was found that the over-expression of the *GUS* gene could not be detected in the Sf9 cells transfected by the shrimp IHHNV-based expression vector of pUC19-IHHNV-PH-GUS alone; that is, no blue signal could be observed in the transfected Sf9 cells after X-gluc staining. The sequencing result confirmed that the construction of the above-mentioned expression vector of pUC19-IHHNV-PH-GUS was successful. Thus, we think the specific transcriptional activation pattern for the insect baculovirus (*Autographa californica* multicapsid nucleopolyhedrovirus, AcMNPV) late promoter of PH (polyhedron) might contribute to a great degree to the failure of the over-expression of the *GUS* gene in the pUC19-IHHNV-PH-GUS-transfected Sf9 cells.

To test our hypothesis and to improve the transfection and expression efficiencies of pUC19-IHHNV-PH-GUS in the Sf9 cells, two viral expression plasmids of pUC19-IHHNV-PH-GUS and Bacmid were co-transfected into the Sf9 cells. It was found that the GUS expression signals (i.e., blue cells) were successfully detected in the co-transfected Sf9 cells, as expected (Figure 3(B2–F2,B3–D3)). In consideration of the fact that high transfection and expression efficiency is very important for efficient viral packaging, the co-transfection ratio of pUC19-IHHNV-PH-GUS to Bacmid (by mass, μg) and the transfection reagent ratio of Cellfectin II (μL) per μg plasmid DNAs were further optimized. As shown in Figure 3(B2–F2), it was found that the best transfection and expression efficiency was obtained when the co-transfection ratio of the above-mentioned two plasmids was 1:1. Also, the optimal transfection reagent ratio was 6 μL Cellfectin II per μg total plasmid DNA (Figure 3(B3–D3)). In a word, the optimal transfection and packaging conditions for pUC19-IHHNV-PH-GUS and Bacmid in Sf9 cells had been developed.

In consideration of the low tropism of Bacmid in shrimp cells, as reported previously by Wu et al. [9], the co-transfection of pUC19-IHHNV-PH-GUS and Bacmid-VP28 was also tested using the previously optimized co-transfection condition in this study. As shown in Figure 3(A4,B4), strong GUS expression signals were detected in the co-transfected Sf9 cells, suggesting a significantly higher transfection and expression efficiency when the recombinant baculovirus expression plasmid of Bacmid-VP28 was used instead of Bacmid in the co-transfection assay. 

### 2.4. Verification of the Packaging Capacity of Shrimp IHHNV-Based Expression Vector of pUC19-IHHNV-PH-GUS into Infective Virions in Sf9 Cells

**Figure 4:**
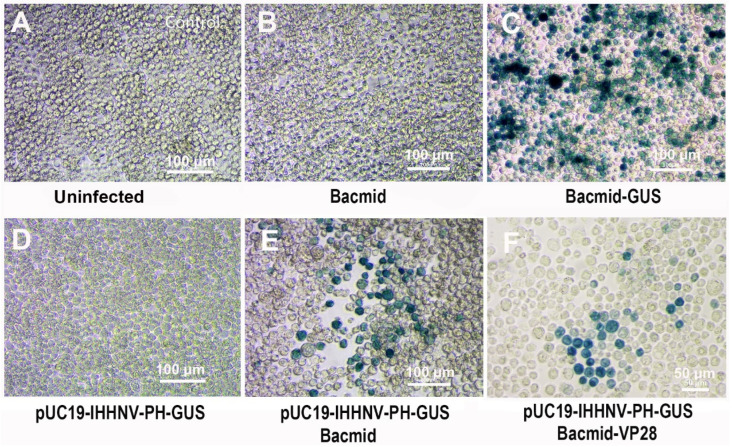
Verification of the packaging capacity of the shrimp IHHNV-based expression vector of pUC19-IHHNV-PH-GUS in Sf9 cells by reinfection assay. All the infected Sf9 cells were stained by X-gluc at 120 h post infection. (**A**) Control Sf9 cells treated by the medium supernatant of un-transfected Sf9 cells. (**B**) Sf9 cells infected by the medium supernatant of Bacmid-transfected Sf9 cells. (**C**) Sf9 cells infected by the medium supernatant of Bacmid-GUS-transfected Sf9 cells. (**D**) Sf9 cells infected by the medium supernatant of pUC19-IHHNV-PH-GUS-transfected Sf9 cells. (**E**) Sf9 cells infected by the medium supernatant of co-transfected Sf9 cells by pUC19-IHHNV-PH-GUS and Bacmid. (**F**) Sf9 cells infected by the medium supernatant of co-transfected Sf9 cells by pUC19-IHHNV-PH-GUS and Bacmid-GUS. Scale bar, 100 μm in panels (**A**–**E**) and 50 μm in panel (**F**).

The over-expression of the shrimp IHHNV-based expression vector of pUC19-IHHNV-PH-GUS in Sf9 cells had been confirmed when it was co-transfected with an insect baculovirus vector of Bacmid or Bacmid-VP28. But it was still unknown whether or not the shrimp IHHNV-based expression vector of pUC19-IHHNV-PH-GUS could be packaged into an infectious virion in Sf9 cells. To solve this problem, a reinfection assay was first carried out. As shown in Figure 4, a lot of blue Sf9 cells could be observed in the Sf9 cells infected by the viral supernatant of co-transfected Sf9 cells by pUC19-IHHNV-PH-GUS and Bacmid (or Bacmid-GUS), indicating that the shrimp IHHNV-based expression vector of pUC19-IHHNV-PH-GUS had been successfully packaged into infective virions (i.e., viral particles) in the Sf9 cells (Figure 4E,F). In contrast, no blue cells could be detected in the uninfected and Bacmid (negative control)-infected Sf9 cells (Figure 4A,B), and many more blue cells could be found in the Bacmid-GUS (positive control)-infected Sf9 cells (Figure 4C). No blue cells could be observed in the Sf9 cells infected by the viral supernatant of pUC19-IHHNV-PH-GUS-transfected Sf9 cells, too. However, based on the fact that the activity of the PH promoter is Bacmid-dependent, from this negative result of the reinfection assay in the single plasmid of pUC19-IHHNV-PH-GUS-transfected Sf9 cells, we still did not know if the viral vector of pUC19-IHHNV-PH-GUS could be successfully packaged into infective virions.

**Figure 5:**
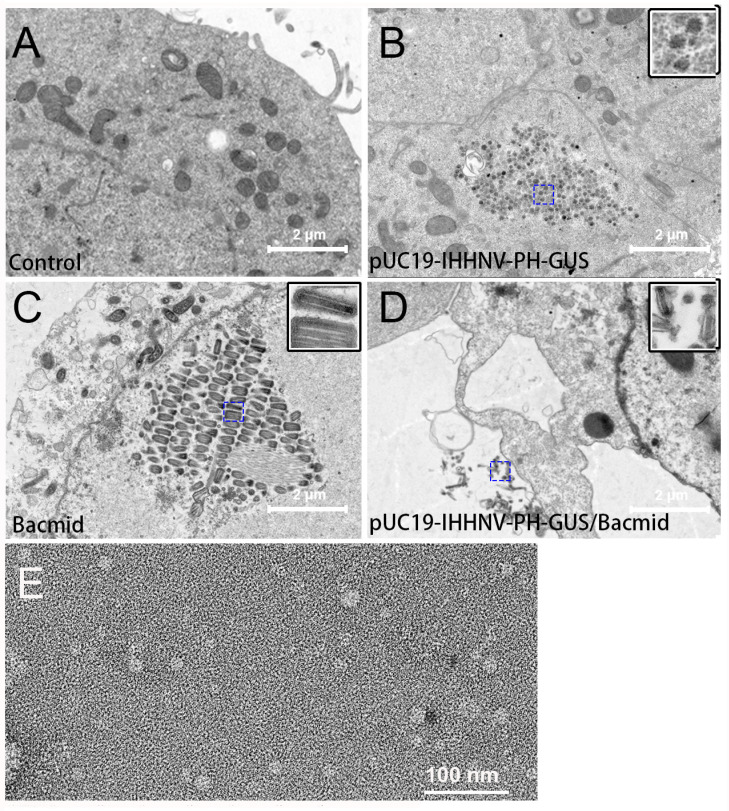
Verification of the packaging capacity of the shrimp IHHNV-based expression vector of pUC19-IHHNV-PH-GUS into infective virions in Sf9 cells by electron microscopy assay. Panels **A(**–**D**) are the results of the transmission electron microscopy assay. Panel (**E**) is the result of the electron microscopy negative staining assay. (**A**) Uninfected Sf9 cells without any virions detected. (**B**) Sf9 cells reinfected by the medium supernatant of pUC19-IHHNV-PH-GUS-transfected Sf9 cells, only IHHNV-like virions could be observed. (**C**) Sf9 cells reinfected by the medium supernatant of Bacmid-transfected Sf9 cells, only Bacmid virions could be found. (**D**) Sf9 cells reinfected by the medium supernatant of the co-transfected Sf9 cells by pUC19-IHHNV-PH-GUS and Bacmid, and both IHHNV-like and Bacmid virions could be detected. (**E**) The packaged virions of pUC19-IHHNV-PH-GUS detected from the medium supernatant of pUC19-IHHNV-PH-GUS-transfected Sf9 cells. The insets in panels (**B**–**D**) are enlargements of the blue square box in the dotted line. Scale bars were 2 μm in Panels (**A**–**D**) and 100 nm in Panel (**E**).

The packaging capacity of the shrimp IHHNV-based expression vector of pUC19-IHHNV-PH-GUS in Sf9 cells was further verified by electron microscopy observation. As shown in Figure 5, transmission electron microscopy analysis of the Sf9 cells infected by the medium supernatant of pUC19-IHHNV-PH-GUS-transfected Sf9 cells indicated that there were a plenty of IHHNV-like icosahedral particles piled up, in aggregates or separately, in the infected Sf9 cells (Figure 5B). In contrast, no viral particles could be detected in the uninfected Sf9 cells (Figure 5A). Only Bacmid virions could be found to gather and line up in rows in the Sf9 cells infected by the medium supernatant of Bacmid-transfected Sf9 cells (Figure 5C). Both IHHNV-like and Bacmid virions could be observed in the Sf9 cells infected by the medium supernatant of pUC19-IHHNV-PH-GUS and Bacmid-co-transfected Sf9 cells (Figure 5D), suggesting that the insect Sf9 cells co-transfected with two viral vectors of pUC19-IHHNV-PH-GUS and Bacmid were able to package two kinds of viruses simultaneously. As shown in Figure 5E, the results of the electron microscopy negative staining analysis confirmed that IHHNV-like viral particles of pUC19-IHHNV-PH-GUS could be detected from the medium supernatant of pUC19-IHHNV-PH-GUS-transfected Sf9 cells, with a similar size to the wild-type shrimp IHHNV, which had been reported to be an icosahedral viral particle with an average diameter of 22–27 nm [18,24,35]. 

Taken together, the results obtained from the reinfection and electron microscopy analysis confirmed the packaging capacity of the insect Sf9 cells to produce infectious shrimp IHHNV-like viral particles after transfection of the viral vector of pUC19-IHHNV-PH-GUS.

### 2.5. Infection and GUS Expression in Shrimp Hemolymph Cells by Mixed Viruses of pUC19-IHHNV-PH-GUS and Bacmid (or Bacmid-VP-28)

**Figure 6:**
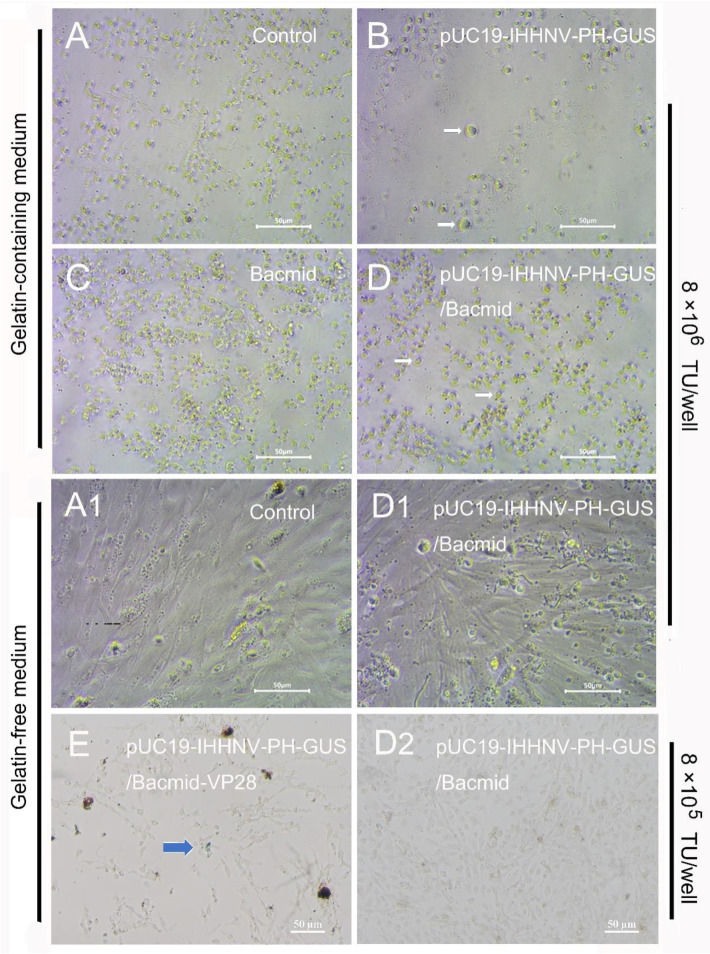
Infection and GUS expression of the mixed viruses of pUC19-IHHNV-PH-GUS and Bacmid (or Bacmid-VP-28) in the primarily cultured shrimp hemolymph cells cultured in gelatin-containing 1 × L-15-based medium or gelatin-free 1.5 × L-15-based medium. The GUS expression was detected by X-gluc staining at the 5th day post infection. (**A**,**A1**) Uninfected cells. (**B**) Cells infected by IHHNV-like viruses of pUC19-IHHNV-PH-GUS. (**C**) Cells infected by the baculovirus of Bacmid. (**D**,**D1**,**D2**) Cells infected by varied doses of mixed viruses of pUC19-IHHNV-PH-GUS and Bacmid but cultured in gelatin-containing (**D**) or gelatin-free (**D1**,**D2**) medium. (**E**) Cells infected by mixed viruses of pUC19-IHHNV-PH-GUS and Bacmid-VP28 and cultured in gelatin-free medium. The white arrows show the cell hypertrophy. The blue arrow indicates the positive cells with GUS expression. Scale bar, 50 μm.

First, the mixed virus of pUC19-IHHNV-PH-GUS and Bacmid was successfully packaged and titrated in Sf9 cells, and the titer of the mixed viruses packaged was up to 4 × 10^8^ TU/mL after purification and concentration. However, as shown in Figure 6, when this kind of mixed virus was used to infect the shrimp hemolymph cells at an infection dose of 8 × 10^6^ TU/well in a 96-well culture plate, no GUS expression or blue cells could be observed in the infected shrimp hemolymph cells, no matter whether they were cultured in the gelatin-containing 1 × L-15-based medium or gelatin-free 1.5 × L-15-based medium (Figure 6(D,D1)). The extremely low tropism of the baculovirus of Bacmid in shrimp hemolymph cells might have contributed to a great degree to the failure of the GUS expression of the mixed viruses of pUC19-IHHNV-PH-GUS and Bacmid [10]. In other words, the baculovirus of Bacmid failed to infect and enter into the shrimp hemolymph cells; thus, it could not activate the PH promoter activity of the IHHNV-based vector of pUC19-IHHNV-PH-GUS and eventually resulted in the failure of GUS expression within the shrimp hemolymph cells. In the uninfected, Bacmid-infected and single virus of pUC19-IHHNV-PH-GUS-infected shrimp hemolymph cells, as expected, no GUS expression or blue cells were detected. Noteworthily, obvious cell hypertrophy was detected in the pUC19-IHHNV-PH-GUS-infected shrimp cells (Figure 6B), and slight cell hypertrophy was observed in the mixed virus-infected shrimp cells instead (Figure 6D), both cultured in gelatin-containing 1 × L-15-based medium. The reasons for the occurrence of cell hypertrophy in the infected shrimp cells were uncertain. 

To improve the tropism and infectivity of Bacmid in shrimp cells, an alternative recombinant baculovirus vector of Bacmid-VP28 was used to produce the mixed viruses of pUC19-IHHNV-PH-GUS and Bacmid-VP28 by the co-transfection method in Sf9 cells. The titer of the above-mentioned mixed virus was up to 2 × 10^8^ IU/mL after purification and concentration, which was only half of the obtained titer of mixed viruses of pUC19-IHHNV-PH-GUS and Bacmid. As shown in Figure 6E, when the mixed viruses of pUC19-IHHNV-PH-GUS and Bacmid-VP28 were used to infect the shrimp hemolymph cells cultured in gelatin-free 1.5 × L-15-based medium at an infection dose of 8 × 10^5^ TU/well in a 96-well culture plate, the GUS expression or blue cells were successfully observed in the infected shrimp hemolymph cells, but in a very low infection and expression efficiency, suggesting the higher infection efficiency of the recombinant baculovirus Bacmid-VP28 in the shrimp hemolymph cells. In other words, the improved GUS expression efficiency could be attributed to the use of Bacmid-VP28 and the driving activity of the PH promoter is highly dependent on the presence of Bacmid-VP28. 

### 2.6. Infection and GUS Expression in Adult Shrimps by Mixed Viruses of pUC19-IHHNV-PH-GUS and Bacmid (or Bacmid-VP-28)

**Figure 7:**
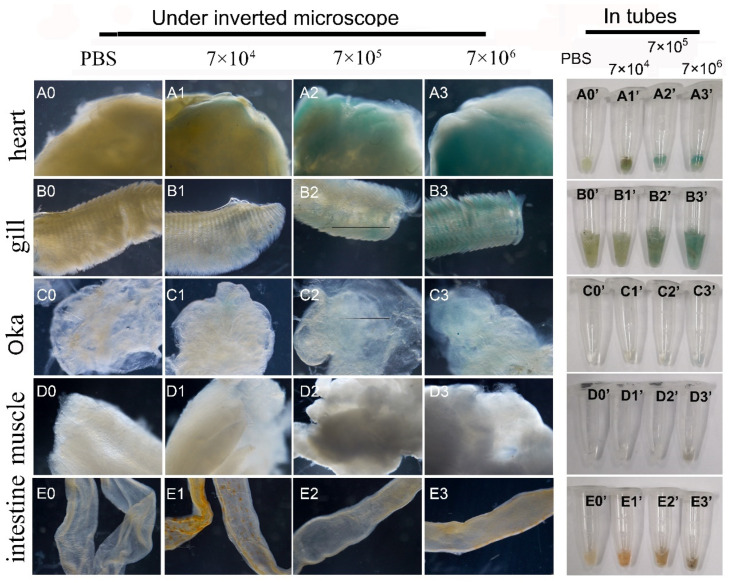
Infection and GUS expression analysis in different tissues of adult shrimps infected by varied doses of mixed viruses of pUC19-IHHNV-PH-GUS and Bacmid. The sampled adult tissues of the infected shrimps were first stained by X-gluc in a 1.5 mL centrifuge tube (right) and then observed under an inverted phase contrast microscope (left). (**A0**–**E0**) and (**A0’**–**E0’**) Results of the hearts, gills, Oka organs, muscles and intestines of the shrimps after injection of PBS, respectively. (**A1**–**E1**) and (**A1’**–**E1’**) Results of the five tested tissues of the shrimps after injection of 7 × 10^4^ TU mixed viruses, respectively. (**A2**–**E2**) and (**A2’**–**E2’**) Results of the five tested tissues of the shrimps after injection of 7 × 10^5^ TU mixed viruses. (**A3**–**E3**) and (**A3’**–**E3’**) Results of the five tested tissues of the shrimp after injection of 7 × 10^6^ TU mixed viruses.

First, the mixed viruses of pUC19-IHHNV-PH-GUS and Bacmid was intramuscularly injected into the second abdominal segment of the adult shrimps at varied infection doses of 7 × 10^4^, 7 × 10^5^ and 7 × 10^6^ TU per shrimp. Five days later, to detect the expression of the *GUS* gene, the tissues of the hearts, gills, Oka organs, muscles and intestines of the injected shrimps were dissected, respectively, and first stained by X-gluc in a 1.5 mL centrifuge tube and then photographed under an inverted phase contrast microscope. As shown in Figure 7, it was found that GUS expression could be detected in the tissues of most of the injected shrimps, with an infection efficiency of 70–80%, and showed an obvious tissue-specificity. In detail, a strong blue signal could be detected in the hearts and gills, but a weak blue signal in Oka organs and no blue signal in the intestines and muscles after X-gluc staining. Moreover, the intensity of the blue signals in the hearts, gills and Oka organs increased with the increase of the infection doses, showing an obvious dose-dependent relation. For example, a weak blue signal could be observed in the Oka organs only at the highest infection dose of 7 × 10^6^ TU per shrimp. Noteworthily, there was a visible background blue signal in the hearts and gills of the PBS-injected control shrimps, showing a tissue-specific background GUS expression (Figure 7(A0,A0’,B0,B0’)).

**Figure 8:**
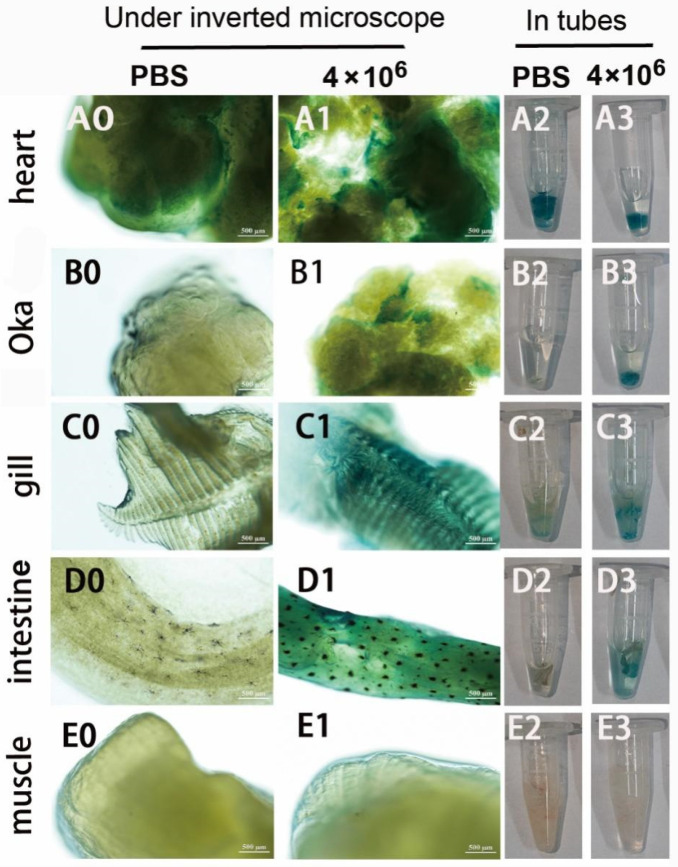
Infection and GUS expression analysis in different tissues of adult shrimps infected by mixed viruses of pUC19-IHHNV-PH-GUS and Bacmid-VP28. The sampled adult tissues of the infected shrimps were first stained by X-gluc in a 1.5 mL centrifuge tube (right) and then observed under an inverted phase contrast microscope (left). (**A0**–**E0**) and (**A2**–**E2**) Results of the hearts, Oka organs, gills, intestines and muscles of the shrimp after injection of PBS, respectively. (**A1**–**E1**) and (**A3**–**E3**) Results of the hearts, Oka organs, gills, intestines and muscles of the shrimp after injection of 4 × 10^6^ TU mixed viruses, respectively. Scale bar, 500 μm.

Second, the infection and GUS expression capability of the mixed viruses of pUC19-IHHNV-PH-GUS and Bacmid-VP28 in the adult tissues of shrimps were further examined by intramuscular injection at a relatively lower infection dose of 4 × 10^6^ TU per shrimp. As shown in Figure 8, it was found that, unlike the mixed viruses of pUC19-IHHNV-PH-GUS and Bacmid, strong blue signals could be detected not only in the hearts and gills but also in the Oka organs and intestines of the shrimps infected by the mixed viruses of pUC19-IHHNV-PH-GUS and Bacmid-VP28, suggesting a much higher infection and GUS expression capability of the mixed viruses when Bacmid-VP28 was included. One possible explanation for this is that Bacmid-VP28 had much higher infectivity in shrimp tissues than Bacmid did. Similarly, no blue signal could be detected in the muscles of the infected shrimps, inferring that muscle tissue was the most difficult tissue to be infected or that a higher infection dose was needed. It could also be noted that an obvious background blue signal was detected in the hearts, while weaker but visible background blue signals were observed in the gills, Oka organs and intestines. 

**Figure 9:**
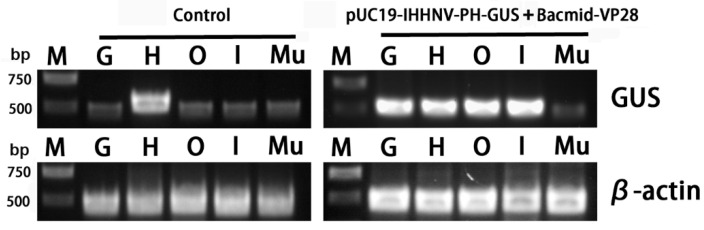
Semi-quantitative RT-PCR analysis of the *GUS* gene expression in different tissues of the adult shrimps uninfected (control) and infected by mixed viruses of pUC19-IHHNV-PH-GUS and Bacmid-VP28. M, Trans 2K plus DNA marker. G, gill. H, heart. O, Oka organ. I, intestine. Mu, muscle. GUS, β-glucuronidase gene. β-actin, internal reference gene, from shrimp (*P. vannamei*).

Finally, to confirm the infection and over-expression of the *GUS* gene in the tested tissues, semi-quantitative RT-PCR analysis was performed. It was found that the heart tissues had a strong background GUS expression level, whereas the other four tested tissues of the gills, Oka organs, intestines and muscles had a relatively low background GUS expression level (Figure 9). This is reasonable because the *GUS* gene encodes a kind of hydrolytic enzyme of β-glucuronidase, which can degrade glucuronide esters [36,37]. Although there was a strong background GUS expression level in the hearts, the semi-quantitative RT-PCR analysis results could still confirm the successful infection and over-expression of the *GUS* gene in the other tissues of the gills, Oka organs and intestines of the shrimps infected by the mixed viruses of pUC19-IHHNV-PH-GUS and Bacmid-VP28 (Figure 9).

## 3. Discussion

In this study, the genomic DNA of the shrimp IHHNV had been successfully isolated and inserted into a prokaryotic cloning plasmid of pUC19-IHHNV. This made it possible for the genomic DNA of a shrimp virus to be multiplied in bacterial cells. Subsequently, a shrimp IHHNV-based expression vector of pUC19-IHHNV-PH-GUS was constructed by introducing a eukaryotic expression cassette of PH-GUS-MCS-SV40 pA into the aforementioned cyclized plasmid immediately after the IHHNV genome. It was also found that insect Sf9 cells could be used as a packaging cell line for the shrimp IHHNV-based expression vector of pUC19-IHHNV-PH-GUS, as confirmed by the reinfection, transmission electron microscopy and electron microscopy negative staining assays. Shrimp IHHNV-like icosahedral virus particles with a similar size to wild-type IHHNV could be observed both in the Sf9 cells and in the medium supernatant of pUC19-IHHNV-PH-GUS-infected Sf9 cells. 

The PH promoter was chosen in the IHHNV-based expression vector of pUC19-IHHNV-PH-GUS because it has a strong activity and can produce high-level protein expression in Sf9 cells, thus proving beneficial for the viral packaging. However, as it is a late-stage promoter of insect baculovirus, its activation is dependent on the expression products of some early genes of baculovirus [38,39,40]. The absence of Bacmid (genetically modified from AcMNPV) might result in the over-expression failure of the PH promoter-driven *GUS* gene. As expected, no GUS expression or blue signal could be detected in the single plasmid of the pUC19-IHHNV-PH-GUS-transfected Sf9 cells, but many blue cells could be detected in the co-transfected Sf9 cells by pUC19-IHHNV-PH-GUS and Bacmid (or Bacmid-VP28). Moreover, a significantly higher transfection and expression efficiency was obtained when the baculoviral plasmid of Bacmid-VP28 encoding the shrimp WSSV-sourced envelope protein VP28 was used instead of Bacmid. 

It is well known that the host range (i.e., tropism) of an enveloped virus is usually dependent on its outermost envelope proteins, which can recognize and bind the shrimp cell surface receptors and mediate the viral entry into the host cells [41]. The recombinant baculovirus of Bacmid-VP28 encoded an important envelope protein of VP28 from shrimp white spot syndrome virus (WSSV), which can bind to shrimp cells as an attachment protein and help the virus enter the cytoplasm, and it had been confirmed that the introduction of the VP28 protein into the envelope of pseudo-typed baculovirus, lentivirus and retrovirus could significantly improve their tropism to shrimp cells [4,6,10]. Thus, it can be expected that the baculovirus of Bacmid-VP28 will have higher tropism in shrimp cells than Bacmid. In this study, when the mixed viruses of pUC19-IHHNV-PH-GUS and Bacmid (or Bacmid-VP28) were used to infect the primarily cultured shrimp hemolymph cells, no GUS expression or blue cells could be detected after infection by the mixed viruses of pUC19-IHHNV-PH-GUS and Bacmid, possibly due to the extremely low tropism and infectivity of Bacmid in the shrimp hemolymph cells. Instead of this, obvious blue cells were observed in the shrimp hemolymph cells infected by the mixed viruses of pUC19-IHHNV-PH-GUS and Bacmid-VP28, possibly due to the higher infectivity and cell entry of Bacmid-VP28 in shrimp hemolymph cells. In a word, the infection and GUS expression capability of the mixed viruses could be greatly improved when Bacmid-VP28 was used to replace Bacmid. In addition, the prerequisite for GUS expression was the successful co-infection and cell entry of the two mixed viruses of pUC19-IHHNV-PH-GUS and Bacmid-VP28 into the same shrimp hemolymph cell. However, the co-infection efficiency of the above-mentioned mixed viruses in shrimp hemolymph cells was still unclear and more work is needed on it. Anyway, the use of a higher infection dose should be a better choice to increase the infection and expression efficiency of the mixed viruses of pUC19-IHHNV-PH-GUS and Bacmid-VP28 in the shrimp hemolymph cells. 

Unlike shrimp hemolymph cells, both of these two kinds of mixed viruses could infect the adult tissues of most of the injected shrimps with an infection efficiency of 70–80% and in tissue-specific and dose-dependent manners, and this was confirmed by semi-quantitative RT-PCR analysis. One possible reason for the significantly higher infection efficiencies of the above-mentioned two kinds of mixed viruses is the higher dividing capacity of live shrimp tissues in comparison with the in vitro cultured shrimp cells. Of note, unlike the mixed viruses of pUC19-IHHNV-PH-GUS and Bacmid, strong blue signals could be detected not only in the hearts and gills but also in the Oka organs and intestines of the shrimps infected by the mixed viruses of pUC19-IHHNV-PH-GUS and Bacmid-VP28, suggesting the much higher infection and GUS expression capability of the mixed viruses when Bacmid-VP28 was included, confirming that Bacmid-VP28 had much higher infectivity in shrimp tissues than Bacmid did.

In addition, VP28 is the most abundant major envelope protein of WSSV, but it is not the only one. In fact, the WSSV envelope consists of at least 35 different proteins, and more than 23 of them are denoted as envelope proteins (GenBank No. AF332093.3). The high diversity of envelope proteins in WSSV may to a great degree contribute to its wide tissue distribution; in other words, WSSV can be detected in almost all the tissues of the infected shrimps. Thus, we think the introduction of other kinds of WSSV envelope proteins into the recombinant baculovirus might change its tissue-specific tropism and confer a crucial factor to influence the tissue-specific tropism of the above mixed viruses [42].

Taken together, a shrimp virus (IHHNV)-mediated gene transfer and expression system was successfully developed in this study, which consisted of a shrimp IHHNV-based expression vector of pUC19-IHHNV-PH-GUS, a helper baculovirus of Bacmid-VP28 and one packaging cell line of insect Sf9. This useful research tool for efficient gene transfer and expression in shrimps will greatly promote future works on the molecular breeding of transgenic shrimps with merit traits as well as the immortalization of the in vitro cultured shrimp cells, although it still needs more improvements in terms of the gene delivery efficiency and biosafety assessment, for example, the use of IHHNV with full-length 5′ and 3′ ITR, or the production of replication-defective IHHNV by splitting up of the capsid gene (ORF3) from the IHHNV genomic DNA. 

## 4. Materials and Methods

### 4.1. Shrimps

Actively swimming shrimps (*Metapenaeus ensis*), 12 ± 3 cm in length and 15 ± 5 g in weight, were purchased from a local seafood market in Nanshan, Qingdao, China. They were acclimatized in aerated seawater at an ambient temperature of 18–25 °C for at least two days before being used for viral infection and primary cell culture. The infectious hypodermic and hematopoietic necrosis virus (IHHNV)-infected shrimps were sampled from the local shrimp farm in Qingdao, China, immediately frozen and transported in liquid nitrogen to laboratory, and then stored at −80°C until used for the isolation of the genomic DNA of IHHNV. The care and use of the shrimps were performed under supervision and approved by the scientific ethics committee of Ocean University of China.

### 4.2. Cells and Cell Culture

A continuous insect cell line of Sf9, derived from the ovarian tissues of the pupa of fall army worm, *Spondoptera frugiperda*, was used for the packaging and titration of the shrimp IHHNV-based recombinant viruses, the baculoviruses of Bacmid (i.e., bMON14272, from the Bac-to-Bac baculovirus expression system, Cat. No. 10360-014, Invitrogen, Carlsbad, CA, USA), Bacmid-GUS encoding β-glucuronidase (GUS), and Bacmid-VP28 encoding the envelope protein (VP28) of shrimp WSSV. The Sf9 cells were maintained in SIM medium (Sino Biological, Beijing, China) supplemented with 10% fetal bovine serum (FBS, Biological Industries, Kibbutz Beit Haemek, Israel, ISR) at 28 °C in a 3% CO_2_ incubator. 

Primarily cultured shrimp hemocytes were isolated from the circulating hemolymph of *M. ensis* and then cultured in a 1.5 × L-15-based or gelatin-containing 1.0 × L-15-based growth medium, as described previously by Han et al. [43] and Zhao et al. [44], respectively, until used for the infectivity analysis of the shrimp IHHNV-based recombinant viruses or the baculoviruses of Bacmid, Bacmid-GUS and Bacmid-VP28. In brief, the shrimps were pre-treated overnight in aerated boiling-disinfected seawater containing 1200 IU/mL penicillin and 1200 IU/mL streptomycin. Then, the shrimps were individually anesthetized by immersion in 75% ethanol for 3–5 min. Next, after sequential disinfection of the body surface of the shrimps by iodophor (INOHV, Qingdao, China) and 75% ethanol, the circulating hemolymphs were drawn out from the thoracic sinus of the shrimp using a 1 mL aseptic injection syringe preloaded with 200 μL 1.5 × L-15-based shrimp growth medium, then mixed and seeded into a 96-well culture plate (100 μL/well) and cultured at 28 °C in a 3% CO_2_ incubator for 4 h. After that, the old medium in each well was replaced with fresh 1.5 × L-15-based or 1 × L-15 gelatin-containing growth medium and cultured until used.

### 4.3. Cloning and Cyclizing of the Genomic DNA of Shrimp IHHNV

The published full-length genomic DNA of shrimp infectious hypodermic and hematopoietic necrosis virus (IHHNV) was 4.1 kb in size, which had two highly variable inverted terminal repeats (ITR) in both ends. After many attempts, only 3833 bp of the length of the genomic DNA of IHHNV, with shortened ITR in both ends, had been cloned in this study. In detail, the genomic DNA of IHHNV was amplified by PCR in two overlapped fragments, 1–1910 bp and 1870–3833 bp, respectively. In order to link them together, these two fragments had a 40 bp overlap located in position 1870–1910 bp, which served as a homology arm for recombinase-based assembly. The genomic DNA of IHHNV was cyclized by head-to-tail joining with a linearized prokaryotic cloning plasmid of pUC19, which was pre-digested with double restriction endonucleases of *Hind* III and *Bam*H I. Thus, an IHHNV-containing shuttle plasmid of pUC19-IHHNV was constructed to be used to multiply the genomic DNA of IHHNV in bacteria. 

To clone and cyclize the genomic DNA of IHHNV, the anterior fragment of 1–1910 bp was amplified by a forward primer of 5′-TCACACAGGAAACAGCTATGACCATGATTACGCCAAGCTTTCGGAGCGCTTCGCAGGAAACCGTTACAA-3′, which had a 40 bp homologous arm (underlined) in the 5′-end corresponding to the 40 bp sequence in the upstream of the *Hind* III restriction endonuclease site in the plasmid of pUC19, and a reverse primer of 5′-GCATATTGTCGTAGTCTGGT-3′. The posterior fragment of 1870–3833 bp was amplified by a forward primer of 5′-GTCACTAATTACAAACCTGCAG-3′ and the reverse primer of 5′-AACGACGGCCAGTGAATTCGAGCTCGGTACCCGGGGATCCCTTCGCAGAAACCGTTAAC-3′, which had a 40 bp homologous arm (underlined) in the 5′-end, too, but this homologous arm corresponded to the 40 bp complement sequence in the downstream of the *Bam*H I restriction endonuclease site in the plasmid of pUC19. Finally, the linearized plasmid of pUC19 and the homology arm-carrying IHHNV fragments of 1–1910 bp and 1870–3833 bp were connected head to tail into one cyclized recombinant plasmid of pUC19-IHHNV using a large fragment homologous recombination kit (Gibson Assembly^®^ Ultra Kit, SGI-DNA, CA, USA) according to the manual’s instructions. In brief, a 40 ng anterior fragment, 40 ng posterior fragment and 50 ng linearized pUC19 were first mixed in a 200 μL PCR tube and then 5 μL GA Ultra Master Mix A (2×) was added and mixed again. After that, the mixture was incubated and run under the following PCR program: 37 °C for 5 min, 75 °C for 20 min, and then decreased to 60 °C at a rate of 0.1 °C per second, then 60 °C for 30 min, and finally decreased to 4 °C at a rate of 0.1 °C per second. Next, 10 μL GA Ultra Master Mix B (2×) was added and run at 45 °C for 15 min. Finally, the ligation product was immediately transformed into the T1 competent bacteria cells for the screening and sequencing of positive clones.

### 4.4. Construction of Shrimp IHHNV-Based Expression Vector of pUC19-IHHNV-PH-GUS

First, an expression cassette of PH-GUS-MCS-SV40 pA, 2383 bp in size, consisting of an insect baculovirus polyhedron (PH) promoter, the *GUS* (β-glucuronidase) gene, a downstream MCS (multiple cloning site) and the SV40 poly (A) signal (pA), was amplified by a forward primer of 5′-TCTGCGAAGGGATCCATCATGGAGATAATTAAAATGA-3′ and a reverse primer of 5′-AGTGAATTCGAGCTCGATCCAGACATGATAAGAT-3′ using a donor plasmid of pFastBac^TM^1-GUS as a template. The pFastBac^TM^1-GUS plasmid was purchased along with the Bac-to-Bac baculovirus expression system (Invitrogen, catalog No. 10359-016 and 10360-014, Carlsbad, CA, USA). For recombinase-based assembly, the above forward primer was augmented with a 15 bp homologous arm in its 5′-end (underlined) corresponding to the 15 bp sequence in the upstream of the *Bam*H I restriction endonuclease site in the plasmid of pUC19-IHHNV, and the above reverse primer was augmented with a 15 bp homologous arm in its 5′-end (underlined), which corresponded to the 15 bp complement sequence in the downstream of the *Sac* I restriction endonuclease site in the plasmid of pUC19-IHHNV, respectively. Next, to construct the shrimp IHHNV-based expression vector of pUC19-IHHNV-PH-GUS, the homologous arm-containing fragment of PH-GUS-MCS-SV40 pA was inserted into the pUC19-IHHNV vector, which had been linearized by *Bam*H I and *Sac* I, using a universal homologous recombination kit (Pro Ligation-Free Cloning Kit, ABM Inc., VAN, Vancouver, BC, Canada) according to the manual’s instruction. In brief, the PH-GUS-MCS-SV40 pA fragment, the linearized plasmid of pUC19-IHHNV and 2 × Pro Ligation-Free Mix were mixed and then incubated at 50 °C for 2 h. Then, the ligation product was immediately transformed into DH5α competent bacterial cells for the screening and sequencing of positive clones. 

### 4.5. Viral Packaging

The packaging of pUC19-IHHNV-PH-GUS, Bacmid and Bacmid-GUS were carried out as described previously by Wu et al. [9] with minor modifications. In brief, Sf9 cells were seeded into a 48-well culture plate at a seeding density of 1 × 10^5^ cells/well at about 16 h ahead of transfection, and when the cell confluency reached 70%, the old medium in each well was replaced with a diluted SIM medium containing 13.5% SIM medium, 1.5% FBS and 85% serum-free Grace medium (Gibco, Carlsbad, CA, USA). After that, for each well, a total of 1.875 μL transfection reagent of Cellfectin II (Gibco, Carlsbad, CA, USA) and 0.375 μg viral plasmid DNA were separately diluted in 25 μL serum-free Grace medium in two tubes and incubated at room temperature for 10 min. After that, they were combined and vortexed and incubated at room temperature for another 20–30 min. Next, the liposome–DNA transfection complex was evenly added into the well drop by drop and cultured at 28 °C for 4 h, and then the transfection reagent-containing medium was replaced with normal serum-containing SIM medium. Then, 120 h later, the expression of the *GUS* reporter gene was assayed by X-gluc staining (Solarbio, Beijing, China) as described previously by Wu et al. [9]. The baculoviruses of Bacmid and Bacmid-GUS were chosen as negative and positive controls, respectively, to verify the expression of the *GUS* gene of the shrimp virus (IHHNV)-based expression vector of pUC19-IHHNV-PH-GUS in the Sf9 cells. Baculoviral plasmids of Bacmid and Bacmid-GUS were prepared according to the manual’s instruction for the Bac-to-Bac baculovirus expression system (Invitrogen, Carlsbad, CA, USA). 

A total of two kinds of mixed viruses of pUC19-IHHNV-PH-GUS and Bacmid (or pUC19-IHHNV-PH-GUS and Bacmid-VP28) were packaged in this study. Compared with the baculovirus of Bacmid, the recombinant baculovirus of Bacmid-VP28 encoded an important envelope protein of VP28 of the shrimp WSSV and thus was reported to have better tropism to shrimp cells [10]. Before packaging, firstly, the optimal co-transfection ratio of pUC19-IHHNV-PH-GUS to Bacmid was examined in a 48-well culture plate. In consideration of the cytotoxicity of plasmid DNAs, the tested co-transfection ratios of the pUC19-IHHNV-PH-GUS (0.375 μg) to Bacmid were 1:1/3 (0.125 μg), 1:1/2 (0.1875 μg), 1:1 (0.375 μg), 1:2 (0.75 μg) and 1:3 (1.125 μg) using a transfection reagent ratio of 5 μL Cellfectin II per μg DNA, as reported by Wu et al. [9]. As shown in Figure 3, it was found that the optimal co-transfection ratios of pUC19-IHHNV-PH-GUS to Bacmid were 1:1 (μg). Secondly, the optimal ratio of transfection reagent to the mixed plasmid DNAs was further examined in a 48-well culture plate with a co-transfection ratio of 1:1 (pUC19-IHHNV-PH-GUS to Bacmid in μg). The tested ratios of the mixed plasmid DNAs (μg) to Cellfectin II (μL) were 1:4, 1:5 and 1:6, respectively, based on the work by Wu et al. [9]. 

Finally, the mixed viruses of pUC19-IHHNV-PH-GUS and Bacmid (or pUC19-IHHNV-PH-GUS and Bacmid-VP28) were first packaged under the optimized co-transfection ratio and transfection reagent ratio in a 24-well culture plate. In detail, 1 μg pUC19-IHHNV-PH-GUS, 1 μg Bacmid (or Bacmid-VP28) and 12 μL transfection reagent of Cellfectin II were used in the co-transfection of Sf9 cells. Then, at 120 h post co-transfection, the medium containing detached Sf9 cells and cell debris was collected and then clarified twice by centrifugation at 12,000× *g*, 4 °C for 15 min, and finally, the medium supernatant of the mixed viruses was saved and used to further infect the Sf9 cells cultured in 10 cm petri dishes by 400 μL viral supernatant per dish for the purpose of the multiplication of the mixed viruses. 

### 4.6. Purification, Concentration and Titration of the Viruses

To purify the single virus of pUC19-IHHNV-PH-GUS, the transfected Sf9 cells were collected by trypsinization and centrifugation at 2000× *g*, 4 °C for 15 min and then frozen and thawed for three rounds. Next, the lysed cells were resuspended in sterile PBS (8.0 g NaCl, 0.2 g KCl, 0.2 g KH_2_PO_4_ and 3 g Na_2_HPO_4_·12H_2_O in 1 L dH_2_O, pH 7.0) and then clarified twice by centrifugation at 12,000× *g*, 4 °C for 15 min. Finally, the viral supernatant was collected and concentrated by ultracentrifugation at 140,000× *g*, 4 °C for 2 h. After ultracentrifugation, the supernatant was discarded and the viral precipitate was resuspended with PBS, aliquoted and stored at −80 °C. 

To purify the mixed viruses of pUC19-IHHNV-PH-GUS and Bacmid (or pUC19-IHHNV-PH-GUS and Bacmid-VP28), the medium supernatant was first collected and saved, and then the co-transfected Sf9 cells were collected by trypsinization and centrifugation and then frozen and thawed for three rounds. Next, the lysed cells were resuspended in the saved medium supernatant and then clarified twice by centrifugation at 12,000× *g*, 4 °C for 15 min. Finally, the viral supernatant was collected and concentrated by ultracentrifugation at 140,000× *g*, 4 °C for 2 h. After ultracentrifugation, the virus precipitate was collected and resuspended with PBS, aliquoted and stored at −80 °C. 

For the viral titration, Sf9 cell monolayers were prepared in a 96-well culture plate at a seeding density of 4 × 10^4^ cells per well at 16 h ahead of viral infection. Then, the tested stock solutions of the mixed viruses were serially diluted tenfold, ranging from 10 to 10^7^, in serum- and antibiotic-free SIM medium. And then the old medium in each well was removed and 100 μL viral dilutions were added into each well. Five hours post-infection, the old medium was replaced with normal SIM medium. At 120 h post infection, the expression of the *GUS* reporter gene was detected by X-gluc staining as described previously by Wu et al. [9]. The virus titers were calculated by the following formula: TU/mL (transduction units per mL) = percentage of positive cell colonies × total cell numbers seeded/volume of viral stock (mL).

In consideration of the failure of the PH promoter to drive the expression of the *GUS* gene in the case of single viral vector transfection, the titration of the single virus of pUC19-IHHNV-PH-GUS was determined by the simultaneous co-transfection and titration of the mixed viruses of pUC19-IHHNV-PH-GUS and Bacmid. The purification, concentration and titration of the single baculoviruses of Bacmid-GUS and Bacmid were carried out as described previously by Wu et al. [9].

### 4.7. Verification of the Packaging Capacity of IHHNV-Based Expression Vector of pUC19-IHHNV-PH-GUS into Infective Virions in Sf9 Cells 

The successful packaging of the shrimp IHHNV-based expression vector of pUC19-IHHNV-PH-GUS in Sf9 cells was verified by the three methods of reinfection, transmission electron microscopy (TEM) and electron microscopy negative staining assay in this study. 

For the reinfection assay, the viral expression plasmid of pUC19-IHHNV-PH-GUS was transfected alone or co-transfected with Bacmid (or Bacmid-VP28) into the Sf9 cells as described previously under the optimized transfection conditions. At 120 h post transfection, the medium supernatant was collected and clarified by centrifugation as described previously and then used to reinfect the Sf9 cells cultured in serum- and antibiotic- free SIM medium by an infection dose of 100 μL medium supernatant tested per well in a 48-well culture plate. The next day, the old medium was replaced with normal SIM medium. At 120 h post infection, the expression of *GUS* gene was detected by X-gluc staining. The successful detection of the GUS signal can verify the successful packaging of the mature virions of pUC19-IHHNV-PH-GUS in Sf9 cells. 

For the TEM and electron microscopy negative staining assay, Sf9 cells were inoculated into a 10 cm petri dish at a density of 6 × 10^6^ cells per dish at 16 h ahead of transfection, and the old medium was replaced with 10 mL of diluted SIM medium containing 1.35 mL SIM, 0.15 mL FBS and 8.5 mL Grace medium when the confluency reached 70%. Then, the plasmids of pUC19-IHHNV-PH-GUS and Bacmid were transfected individually or co-transfected into Sf9 cells using the transfection reagent of Cellfectin II (Gibco, Carlsbad, CA, USA). In brief, in one tube, 15 μg pUC19-IHHNV-PH-GUS plasmid, or 15 μg Bacmid, or a 30 μg mixture of the above two plasmids, and in another tube 90 or 180 μL Cellfectin II, was diluted in 500 μL serum- and antibiotic-free Grace medium, respectively, and incubated at room temperature for 10 min. Then, the two tubes containing DNA or Cellfectin II were combined accordingly, mixed and then incubated at room temperature for another 20–30 min. The transfection complex was added drop by drop into the culture dish and then replaced to normal SIM medium at 4 h post transfection. At 120 h post transfection, the medium supernatant was collected and clarified by centrifugation and used to reinfect the Sf9 cells, which were freshly seeded in another 10 cm petri dish and the medium was replaced with serum- and antibiotic- free SIM medium, with an infection dose of 400 μL medium supernatant per dish. At 120 h post infection, the medium supernatant of the pUC19-IHHNV-PH-GUS-transfected Sf9 cells was collected, clarified and saved at 4 °C for electron microscopy negative staining analysis by Savile Biotechnology (Shanghai, China). Next, two mL of 2.5% glutaraldehyde was added into each dish after the medium has been removed, and the fixed Sf9 cells were removed by a cell scraper and collected by centrifugation. The supernatant was discarded and the cell pellet was carefully resuspended in a new fixative solution and incubated at room temperature for another 30 min and then stored at 4 °C until the TEM assay and photographing by Saville Biotechnology (Shanghai, China). 

### 4.8. Analysis of Infection and GUS Expression of Aforementioned Mixed Viruses in Primarily Cultured Shrimp Hemolymph Cells

The primarily cultured shrimp hemolymph cells in a 96-well culture plate were infected with 100 μL viral solution per well containing 8 × 10^6^ TU viruses of Bacmid (negative control) or pUC19-IHHNV-PH-GUS, or 8 × 10^6^ TU mixed viruses of pUC19-IHHNV-PH-GUS and Bacmid, or 8 × 10^5^ TU mixed viruses of pUC19-IHHNV-PH-GUS and Bacmid-VP28, all diluted in 1.5 × L-15 basic medium. At 4 h post infection, the virus-containing medium was replaced with 1.5 × L-15-based growth medium or gelatin-containing 1 × L-15-based growth medium and cultured at 28 °C for another 120 h. Then, the expression of the *GUS* gene was detected by X-gluc staining and observed under inverted phase contrast microscope (Nikon, Tokyo, Japan). 

### 4.9. Analysis of Infection and GUS Expression of Aforementioned Mixed Viruses in the Adult Tissues of Shrimps

For the infection and GUS expression of the mixed viruses of pUC19-IHHNV-PH-GUS and Bacmid, firstly, the mixed virus stock solution was serially diluted by 10, 10^2^ and 10^3^ folds in PBS, respectively, and then injected into the second abdominal segment of the shrimps by three different doses of 7 × 10^4^, 7 × 10^5^ and 7 × 10^6^ TU per shrimp in an injection volume of 10 μL. The control shrimps were injected by the same volume of PBS instead. After injection, the injection site was immediately pressed with the thumb for several seconds to prevent the viruses and hemolymphs from flowing out. After that, the injected shrimp was put back into the seawater and cultured for another 5 days. On the fifth day, various adult tissues of the heart, gill, Oka organ, muscle and intestine of the injected shrimp were dissected out and the expression of *GUS* gene was detected by X-gluc staining, as described previously by Wu et al. [9], respectively. In detail, the tested tissue was placed in a 1.5 mL centrifuge tube after being washed three times in 2 × PBS, and then incubated in the X-Gluc staining solution containing 1 M sodium phosphate (pH 7.0), 0.5 M Na_2_EDTA (pH 8.0), 10% Triton X-100, 50 mM K_3_Fe (CN)6 and 0.1 M 50 mg/mL X-gluc overnight in a 28 °C incubator, and then the expression of the *GUS* gene was detected by the intensity of the blue color. After that, the stained tissues were further photographed under an inverted phase contrast microscope (Nikon, Japan).

For the infection and expression of the mixed viruses of pUC19-IHHNV-PH-GUS and Bacmid-VP28, only one infection dose of 4 × 10^6^ TU per shrimp in the same injection volume of 10 μL was tested and the expression of the *GUS* gene in the different tissues of the infected shrimps was detected by X-gluc staining as described previously and further confirmed by semi-quantitative RT-PCR analysis using *GUS* gene-specific primer pairs. For the semi-quantitative RT-PCR analysis, first, the total RNAs of the tested adult tissues of the hearts, gills, Oka organs, muscles and intestines of the infected and uninfected shrimps were isolated using the TransZol Up Plus RNA Kit (TransGen Biotech, Beijing, China) and then reversely transcribed into cDNAs using the PrimeScript™ RT reagent Kit (TaKaRa, Beijing, China), respectively, and used as the RT-PCR templates. Then, a 499 bp cDNA fragment of the *GUS* gene was amplified using a forward primer of 5′-GCGTTACAAGAAAGCCGGGC-3′ and a reverse primer of 5′-AGTCAACAGACGCGTGGTTA-3′. RT-PCR was performed in a 20 μL reaction volume, including 10 μL of 2× Hieff PCR Master Mix, 1 μL forward primer (10 μM), 1 μL reverse primer (10 μM), 1 μL cDNA template and 7 μL H_2_O. The RT-PCR reaction was run for 94 °C for 5 min, 30 cycles of 94 °C for 30 s, 58 °C for 30 s and 72 °C for 45 s, one cycle of 72 °C for 10 min. A 438 bp shrimp (*P. vannamei*) β-actin fragment was amplified using a forward primer of 5′-CCCAGAGCAAGCGAGGTA-3′ and a reverse primer of 5′-CGGTGGTCGTGAAGGTGT-3′ and used as an internal reference.

## Data Availability

The original contributions presented in the study are included in the article; further inquiries can be directed to the corresponding author.
